# RETRACTION: Diabetic Foot Wound Ulcers Management by Vacuum Sealing Drainage: A Meta‐Analysis

**DOI:** 10.1111/iwj.70258

**Published:** 2025-02-25

## Abstract

**RETRACTION:** YangL. , ZhaoN. , YangM. , HuangJ. , FuX. , LeiC. , and CaiP. , “Diabetic Foot Wound Ulcers Management by Vacuum Sealing Drainage: A Meta‐Analysis,” International Wound Journal 21, no. 1 (2024): e14390, 10.1111/iwj.14390.37704593 PMC10788591

The above article, published online on 13 September 2023, in Wiley Online Library (http://onlinelibrary.wiley.com/), has been retracted by agreement between the journal Editor in Chief, Professor Keith Harding; and John Wiley & Sons Ltd. Following an investigation by the publisher, all parties have concluded that this article was accepted solely on the basis of a compromised peer review process. The editors have therefore decided to retract the article. The authors did not respond to our notice regarding the retraction.

**RETRACTION:** L. Yang , N. Zhao , M. Yang , J. Huang , X. Fu , C. Lei , and P. Cai , “Diabetic Foot Wound Ulcers Management by Vacuum Sealing Drainage: A Meta‐Analysis,” International Wound Journal 21, no. 1 (2024): e14390, 10.1111/iwj.14390.37704593 PMC10788591

The above article, published online on 13 September 2023, in Wiley Online Library (http://onlinelibrary.wiley.com/), has been retracted by agreement between the journal Editor in Chief, Professor Keith Harding; and John Wiley & Sons Ltd. Following an investigation by the publisher, all parties have concluded that this article was accepted solely on the basis of a compromised peer review process. The editors have therefore decided to retract the article. The authors did not respond to our notice regarding the retraction.

